# Genome-wide survey of the bHLH super gene family in *Brassica napus*

**DOI:** 10.1186/s12870-020-2315-8

**Published:** 2020-03-14

**Authors:** Yun-Zhuo Ke, Yun-Wen Wu, Hong-Jun Zhou, Ping Chen, Mang-Mang Wang, Ming-Ming Liu, Peng-Feng Li, Jin Yang, Jia-Na Li, Hai Du

**Affiliations:** 1 College of Agronomy and Biotechnology, Southwest University, Chongqing, 400715 China; 2 Academy of Agricultural Sciences, Southwest University, Chongqing, 400715 China

**Keywords:** *Brassica napus*, bHLH transcription factor, Root, Gene expression

## Abstract

**Background:**

The basic helix-loop-helix (bHLH) gene family is one of the largest transcription factor families in plants and is functionally characterized in diverse species. However, less is known about its functions in the economically important allopolyploid oil crop, *Brassica napus*.

**Results:**

We identified 602 potential bHLHs in the *B. napus* genome (*BnabHLHs*) and categorized them into 35 subfamilies, including seven newly separated subfamilies, based on phylogeny, protein structure, and exon-intron organization analysis. The intron insertion patterns of this gene family were analyzed and a total of eight types were identified in the bHLH regions of *BnabHLHs*. Chromosome distribution and synteny analyses revealed that hybridization between *Brassica rapa* and *Brassica oleracea* was the main expansion mechanism for *BnabHLHs*. Expression analyses showed that *BnabHLHs* were widely in different plant tissues and formed seven main patterns, suggesting they may participate in various aspects of *B. napus* development. Furthermore, when roots were treated with five different hormones (IAA, auxin; GA_3_, gibberellin; 6-BA, cytokinin; ABA, abscisic acid and ACC, ethylene), the expression profiles of *BnabHLHs* changed significantly, with many showing increased expression. The induction of five candidate *BnabHLHs* was confirmed following the five hormone treatments via qRT-PCR. Up to 246 *BnabHLHs* from nine subfamilies were predicted to have potential roles relating to root development through the joint analysis of their expression profiles and homolog function.

**Conclusion:**

The 602 BnabHLHs identified from *B. napus* were classified into 35 subfamilies, and those members from the same subfamily generally had similar sequence motifs. Overall, we found that BnabHLHs may be widely involved in root development in *B. napus*. Moreover, this study provides important insights into the potential functions of the *BnabHLHs* super gene family and thus will be useful in future gene function research.

## Background

Transcription factor genes are widely distributed in the eukaryotic kingdom and usually contain two different functional domains involved in DNA binding and transcriptional activities [1, 2]. The basic helix-loop-helix (bHLH) transcription factor is characterized by a conserved 50–60 amino acid (aa) sequence responsible for DNA binding. This sequence consists of two main regions, namely the basic and the helix-loop-helix (HLH) regions. The basic region is a 10–15 aa region at the N-terminus of the protein, which functions as a DNA recognition motif and allows the binding of the HLH region [3]. The HLH region, which is composed of two relatively conserved amphipathic helices linked by a divergent loop, is approximately 40 aa long with mamy hydrophobic amino acids, which contributes to its function in the dimerization of HLH regions [4, 5].

The *bHLH* gene family existed in land plants over 400 million years ago and were highly conserved during plant evolution [6]. As one of the largest transcription factor gene families in plants, the number of *bHLH* genes appears to have increased as plants evolved. For example, there was only one *bHLH* gene in *Cyanidioschyzon Merolae* [6], 98 in moss [7], 208 in *Zea mays* [8], 167 in *Arabidopsis* [7], 159 in tomato [9], and 230 in Chinese cabbage [10]. The substantial increase in the number of *bHLH* genes was concomitant with their increased involvement in diverse physiological and developmental processes, with the majority being involved in metabolism and development. For instance, *Arabidopsis AtbHLH045/MUTE* controls sequential cell fate [11]; *SlbHLH22* in tomato promotes early flowering and accelerates fruit ripening [12]; the bHLH transcription factor SPATULA (*SPT*) homologs are required for either carpel development or are involved in endocarp margin development in *Arabidopsis* and *Prunus persica* [13, 14]; the bHLH transcription factor *MYC2* homologs regulate sesquiterpene and artemisinin biosynthesis in various species such as *Aquilaria sinensis* and *Artemisia annua* [15, 16]. Meanwhile, some *bHLHs* are related to abiotic stress response, including cold, drought, and salt stresses. For example, the bHLH transcription factor PHYTOCHROME-INTERACTING FACTOR 4 (*PIF4*) in *Arabidopsis* mediates plantstomatal in response to high temperatures [17]; *StbHLH1* in potato also responds to high temperature by regulating anthocyanin biosynthesis [18]; alternatively, *MdbHLH3* in *Malus domestica* responds to low temperature [19]. *bHLHs* also respond to various hormones. For instance, *Arabidopsis MYC2* is well known for its conserved roles in abscisic acid (ABA), jasmonic acid (JA), and light signaling pathways [20–22]. Moreover, *bHLHs*, e.g., *JAMs* and *TT8* are jasmonate-responsive transcription factors involved in secondary metabolism [23]. *bHLHs* also contribute to Fe homeostasis in *Arabidopsis* and rice [24, 25]. Notably, bHLH proteins tend to function in protein complexes. For example, the *Arabidopsis* bHLH gene, GLABRA3 (*GL3*)/ENHANCER OF GLABRA3 (*EGL3*), is widely known as an epidermal cell fate specification gene and hair root regulator that functions by forming a protein complex with TTG1 (WD40 repeat protein) and WER/GL1 (R2R3-MYB protein) (MBW complex) [26–28].

In this study, we identified 602 *bHLHs* in the important economy crop, *Brassica napus* (*BnabHLHs*), and mapped them to the 19 *B. napus* chromosomes. According to our phylogenetic analysis and gene functions, the *B. napus bHLH* gene family is divided into 35 subfamilies with seven subfamilies being newly identified. Conserved non-bHLH motifs along with intron insertion pattern analyses further support our classification. Chromosome localization combined with synteny analyses revealed the expansion mechanism of *BnabHLH*s in *B. napus*. Expression profile analyses revealed the potential functions of *BnabHLH*s, with a focus on their possible roles in roots. qRT-PCR analysis confirmed the features of the ortholog functional genes by hormone induction in roots.

## Results

### *B. napus* contains a large number of *bHLHs*

Referred to our previously described method [29], we performed BLASTP searches (e values of < 1.0) against the proteome data of the two sequenced *B. napus* genomes (Darmor–*bzh* [30], and Zhongshuang11, ZS11 [31] ecotypes) respectively, using the representative protein sequences of *Arabidopsis* bHLH proteins [7] as queries. We found that the sequence quality (including sequence integrity, sequence number and genome annotation information) from Darmor–*bzh* ecotype was better than those from ZS11 by sequence comparative analyses between these two ecotypes.

As a result, 613 non-redundant putative bHLH encoding genes were obtained from *B. napus* genome (Darmor–*bzh*). Subsequently, the protein sequences of putative genes were examined by ExPASy to ensure that the candidates contained the bHLH domain. Consequently, 11 genes were excluded from our dataset as no bHLH domain was identified by ExPASy analysis. Meanwhile, the sequence information of 65 *BnabHLHs* from Darmor–bzh was corrected by the data from the ZS11 genome (Additional file 1: Table S1). Finally, a total of 602 *BnabHLHs* with relatively complete open reading frames [32] were obtained in this study, accounting for approximately 0.60% of the *B. napus* protein-coding genes. The corresponding proportions in wheat, rice, maize, and *Arabidopsis* are 0.55, 0.47, 0.59, and 0.61%, respectively [16]. The candidate *BnabHLHs* were then named according to their chromosomal distribution (Additional file 1: Table S1). Analysis of BnabHLH physicochemical properties showed that the BnabHLH proteins varied in length from 63 to 1440 aa; their molecular weight ranged from 6.9 (BnabHLH105) to 165 kDa (BnabHLH381); and their isoelectric points ranged from 4.36 (BnabHLH562) to 11.79 (BnabHLH023). Subcellular localization analysis demonstrated that all BnabHLHs were located in the nucleus (Additional file 1: Table S1).

For further comparative analysis across different species, we identified 245 *bHLHs* in the *B. oleracea* genome by the same method (Additional file 2: Table S2). Sequence information of the *bHLHs* in other species like *Arabidopsis*, *B. rapa*, tomato, potato, and rice was obtained from previously published studies [8–10, 16, 33].

### Sequences characteristics of the bHLH domains of BnabHLH proteins

To investigate the aa sequence features, we performed multiple sequence alignment analyses of the 602 bHLH domains of candidate BnabHLHs. The results were visualized using Weblogo online software.

**Fig. 1:**
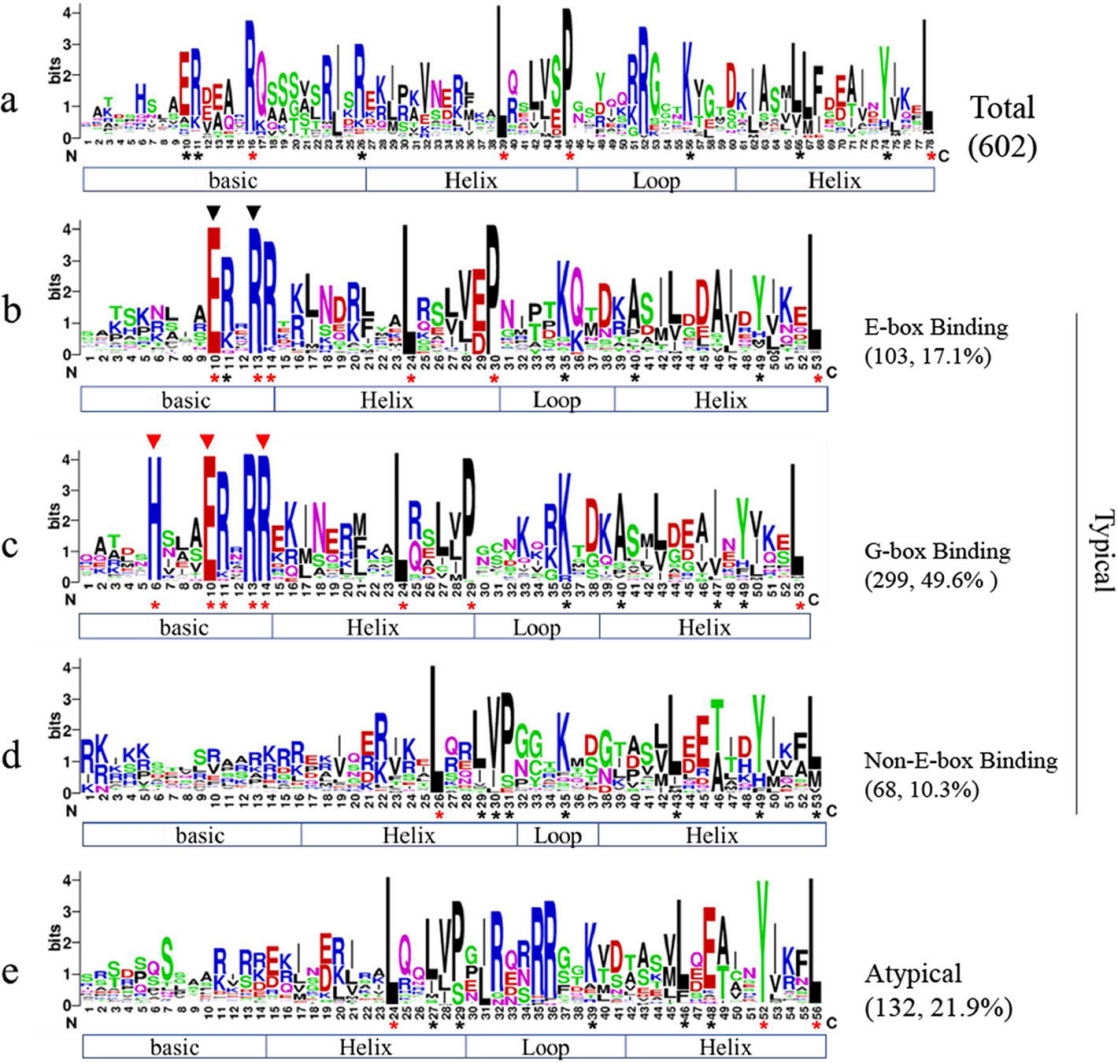
Sequence characteristics of the bHLH domains in different DNA-binding types. Multiple sequence alignments were conducted with the bHLH domains of all candidate proteins and then was conducted with the domains of proteins of a given DNA-binding type. The number in bracket indicates the amount of BnabHLHs in a certain category. Protein secondary structures are illustrated under the sequences. Red asterisks indicate the residues with over 90% similarity; black asterisks indicate residues with over 70% similarity. Black triangles at the top of the sequence indicates the E-box recognition sites; red triangles indicate the G-box recognition sites. The bHLH domains with at least five basic residues but no E-box/G-box binding sites are classified as non-E-box binding genes, otherwise it is a non-DNA binding gene (atypical gene) [6]

Our results showed that the length of the BnabHLH domains was approximately 55 aa, ranging from 39 to 57 aa. The bHLH domain was generally conserved in this gene family in *B. napus*, with ten residues being identified as having a conservation of more than 70% in the bHLH domains (Fig. 1a), including four located in the basic region, five in the two helix regions, and one in the loop region. Consistent with other studies [7–10], Leu-25 was the most conserved residue, with a conservation of almost 100% (Additional file 3: Table S3), indicating its essential role in bHLH proteins. Interestingly, Phe-30 was partly substituted by Ser in *Arabidopsis*, rice, and tomato, among others, however, no such substitution was observed in *B. napus*, *B. oleracea,* or *B. rapa*, (Additional file 3: Table S3), suggesting a higher conservation and/or close relationship in these three species.

To further characterize the BnabHLH sequence features, the criterion given by Massari and Murre [34] was used (Fig. 1). Our results showed that the 602 BnabHLHs were separated into two major categories according to their bHLH domain sequence profiles: 132 (21.9%) atypical BnabHLHs (non-DNA-binding proteins) and 470 (78.1%) typical BnabHLHs (Fig. 1). The latter category was further divided into three categories, including 299 (49.6%) G-box binding proteins, 103 (17.1%) E-box binding proteins, and 68 (10.3%) non-E-box binding proteins (Fig. 1). The sequences of the 132 atypical BnabHLHs were divergent in their basic region but were relatively conserved in their HLH region, especially in the loop region (Fig. 1d). A similar situation was found for the non-E-box binding proteins (Fig. 1c), suggesting its close relationship to the atypical BnabHLHs. In contrast, the residues in the basic region of the E-box/G-box DNA binding proteins were more conserved than the residues in the HLH region (Fig. 1b, c).

### Protein structures of BnabHLHs were conserved in each subfamily

To determine the evolutionary relationship between the *BnabHLHs* from the *Brassicease* species, we constructed an Neighbour-Joining (NJ) phylogenetic tree on the basis of the alignment of 769 bHLH domains from *B. napus* (602) and *Arabidopsis* (167).

**Fig. 2:**
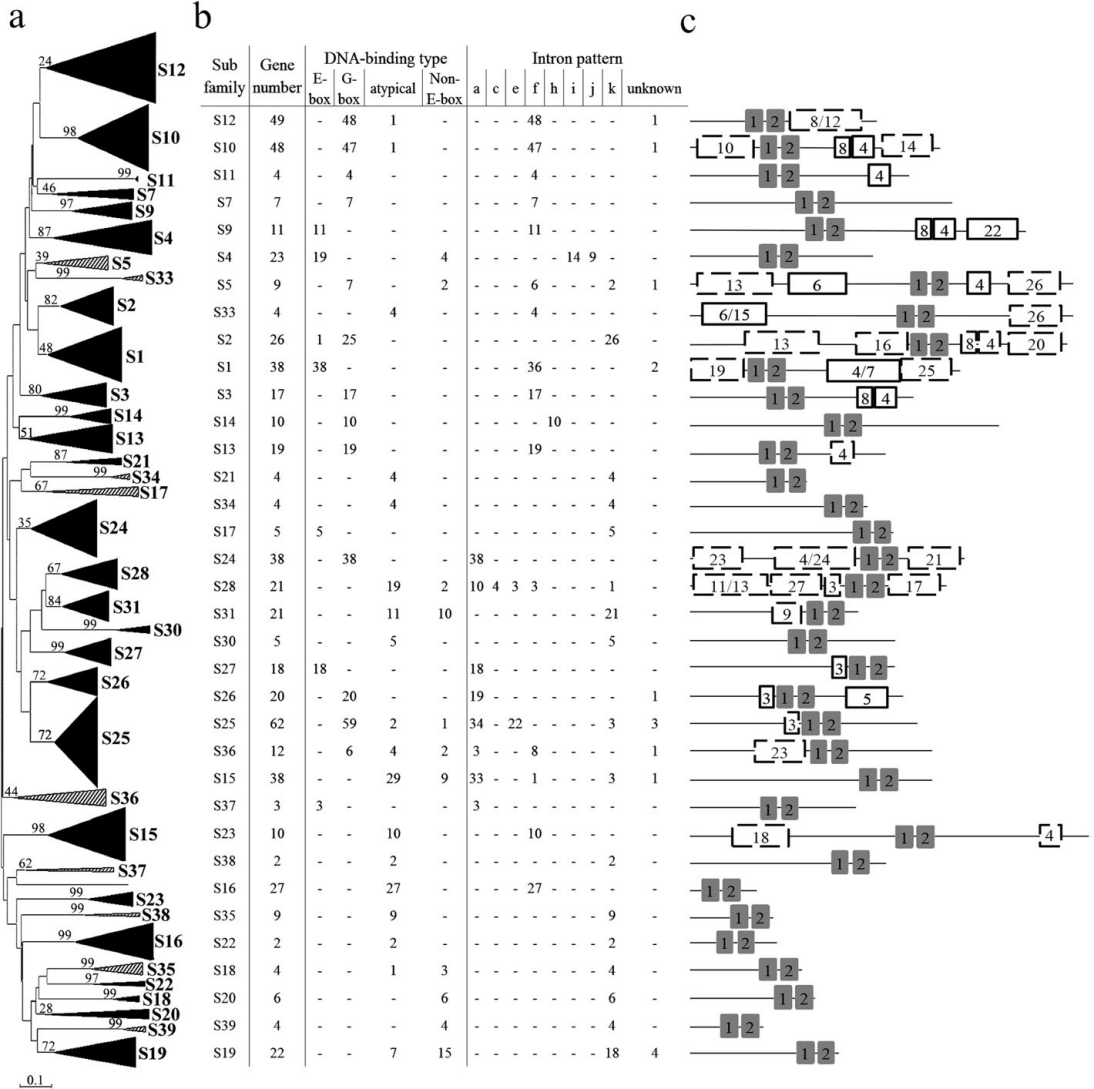
Phylogenetic relationships, DNA-binding types, intron insertion patterns, and architecture of conserved protein motifs in 35 bHLH subfamilies. **a** Phylogenetic relationships of 769 bHLHs from *B. napus* (602) and *Arabidopsis* (167). The phylogenetic tree is generated based on the alignment of bHLH domains of the corresponding bHLH proteins with 1000 bootstrap replicates. The subfamilies marked in grey are newly identified in this study as compared to the results in *Arabidopsis* [7]. **b** DNA-binding types and intron insertion types of *BnabHLHs* in each subfamily. The illustration of intron insertion patterns is shown in Fig. 3. **c** Architecture of conserved protein motifs of BnabHLHs in each subfamily by MEME analysis. Blocks with a black background indicate the bHLH domain. Blocks with a solid line represent the motif distributed in all proteins in a certain subfamily, while those with a dotted line represent the motif distributed in a part of members in a given subfamily

The 769 bHLH proteins were divided into 35 subfamilies, which is the largest number of subfamilies found to date (Fig. 2a). Among these subfamilies, 28 were found previously [7], with seven (S33-S39) being newly identified in this study. Two previously reported subfamilies (S6 and S8) were not found in this study as they were only found in lower plants (moss and algae) [7]. Compared with the division of AtbHLHs, the S5 subfamily in *B. napus* was divided into S5 and S33; S17 was divided into S17 and S34; S21 was divided into S21 and S35; S24 was divided into S24, S36, and S37; S30 was divided into S30 and S38; whereas the orthologs of S39 in *Arabidopsis* were previously defined as orphan genes [7], which were defined as a new subfamily in this study. The distribution of BnabHLHs in the 35 subfamilies was biased, varying from two (S22 and S38) to 62 genes (S25). In addition, the BnabHLHs of different DNA binding types had a biased distribution tendency among different subfamilies as well, but the BnabHLHs in a given subfamily usually shared the same DNA binding type (Fig. 2b). A total of 11 subfamilies (S2, S3, S5, S7, S10, S11, S13, S14, S24, S25, and S26) contained G-box binding proteins; five subfamilies (S1, S9, S17, S27, and S37) contained E-box-binding proteins; three subfamilies (S20, S23, and S39) contained non-E-box-binding proteins; while seven subfamilies (S16, S21, S22, S33, S34, S35, and S38) contained non-DNA-binding proteins (Fig. 2b).

We subsequently used the MEME tool to discover the non-bHLH domains and explore their distribution patterns within each subfamily. A total of 27 conserved motifs of variable lengths (8–103 aa) was obtained from this analysis (Fig. 2c Additional file 4: Table S4). Motifs 1 and 2 were distributed in all BnabHLHs and consisted of the basic and two helix regions of the bHLH domains, respectively. The loop region was located between motif 1 and 2, indicating that this region was more variable than the basic and helix regions. Outside the bHLH domain, members of the same subfamily generally shared several of the same motifs. For example, all BnabHLHs in subfamily S9 contained motif 22; proteins in subfamily S26 all contained motif 5 (Fig. 2b, Additional file 4: Table S4). Moreover, some motifs have been previously characterized and were defined as additional functional motifs. For instance, motifs 4, 8, and 20 were detected in many proteins within subfamilies S2 and S5 in various species, such as TabHLH239, AtMYC2, TabHLH184*,* and ZmbHLH103*,* which were significantly matched with an ACT domain that contributed to the recruitment of the C1 R2R3-MYB factor to the C1 binding sites located in the promoters of flavonoid biosynthetic genes [35]. Meanwhile, motif 6 in these two subfamilies was also conserved, and overlapped with the MIR and MYC_N domains that interact with jasmonate ZIM-domains (JAZs) [36]. Some motifs were subfamily-specific, however their functions are still unclear (Fig. 2c).

### Intron insertion patterns of *BnabHLHs* were conserved within each subfamily

The intron and exon structure is an important clue to understand the gene evolutionary relationship and functional diversification within a gene family [37]. The intron and exon patterns of candidate *BnabHLHs* are determined by comparing their full-length CDS and DNA sequences using the GSDS web server [38].

**Fig. 3:**
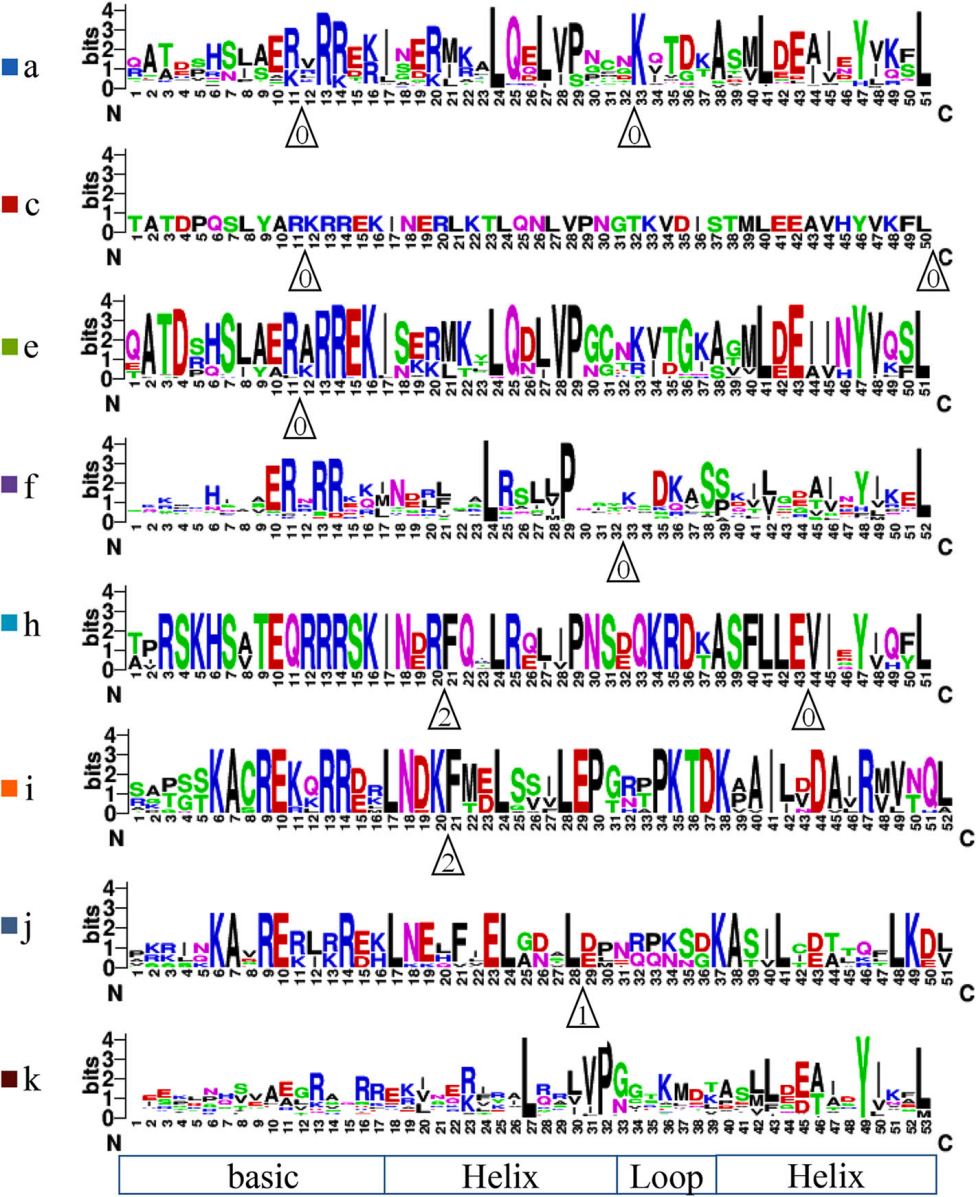
Schematic diagram of intron insertion patterns within the bHLH domains of BnabHLHs. The intron patterns are classified into eight intron types, namely a–k, respectively. Intron insertion sites are indicated by white triangles, and the number within each triangle indicates the splicing phases: 0 refers to phase 0, 1 to phase 1, and 2 to phase 2. A black triangle at the top of a sequence indicates a conserved E-box recognition site. The intron pattern of each subfamily is provided in Fig. 2

A total of eight intron insertion patterns (pattern a to k) were observed in the bHLH domains in *B. napus*, containing 0 to 2 intron insertion sites (Fig. 3). The nomenclature of the intron insertion patterns of *BnabHLHs* is referred to in the study by Carretero-Paulet et al. [7]. In this study, the previously defined pattern a and b (Carretero-Paulet et al., 2010) were characterized as the same type because they share the same insertion sites and phase, and therefore are uniformly named as pattern a. Similarly, pattern d and f (Carretero-Paulet et al., 2010) were uniformly named as f. The intron insertion positions were distributed across the basic and/or HLH regions in the bHLH domain. Among these insertions, those observed in the basic and loop regions were more conserved, while those in the helix regions were variable across different patterns. The intron insertion sites in the basic and helix regions were located at three highly conserved residues, Arg-11 (the E-box recognition site), Phe-21, and Lys-33 (Fig. 3). Furthermore, the intron insertion sites of most patterns were conserved, except pattern j (Fig. 3). Patterns a, c, e, and f were similar, thus they are likely to be homologous, whereas pattern f lacks the first intron as compared with pattern a, pattern e lacks the second intron, and pattern c has the second intron inserted at L-50 as compared with pattern e. A similar situation was observed in patterns h and i. Meanwhile, phylogenetic analyses showed a close relationship between intron insertion patterns a, c, e, and f, further confirming their close relationship. Moreover, patterns k and i appeared to be the ancestral types because they existed in members from algae [7]. Patterns a, f, and k were the three types that accounted for the majority of *BnabHLHs* (41.2, 26.2, and 20.1%, respectively). This trend is similar to the results in other species, such as *Arabidopsis*, rice, potato, poplar, and tomato [8–10, 16, 33]. Accordingly, these three patterns were obtained for many subfamilies while the remaining patterns (i.e., patterns c, e, h, i, and j) existed in only one or two subfamilies, indicating a different expansion trend.

The distributions of intron insertion patterns are generally conserved within most subfamilies. For example, members in subfamilies S12, S10, S11, S7, S9, S5, S33, S2, S1, S13, S23, and S38 contained pattern f, except for several genes that may have been absent due to incomplete genomic annotation information (Fig. 2b). The conservation of intron insertion patterns of *BnabHLHs* within each subfamily provides an independent criterion for the reliability of our phylogenetic analyses (Fig. 2b). Interestingly, the intron insertion patterns of *BnabHLHs* was almost the same as their orthologs in *Arabidopsis*. The only exception was in the S27 subfamily which contains pattern a for *B. napus* members while their homologs in *Arabidopsis* is pattern f [7]. We further compared these results to other species, e.g. rice [7], and found that it should be pattern a for the homologs in this subfamily, including *Arabidopsis* homologs (*At080, At081, At122, At128, At129,* and *At130*).

Overall, intron insertion patterns in *BnabHLHs* were conserved within most subfamilies and corresponded to *AtbHLH* orthologs as well. Moreover, the intron insertion sites in the basic and loop regions were more conserved than those in the helix regions.

### Syntenic analyses revealed duplication events and the expansion mechanism of *BnabHLHs*

In this study, up to 602 *BnabHLHs* were identified, which is significantly higher than the gene number in lower plants, like *Volvox carteri* which only has three [6]. This indicates that there was a large-scale expansion of this gene family that occurred during plant evolution. To explore the expansion mechanism of this gene family in *B. napus*, the chromosomal locations and syntenic relationships of *BnabHLHs* were analyzed based on the genome information from Genoscope and CoGe databases [39].

**Fig. 4:**
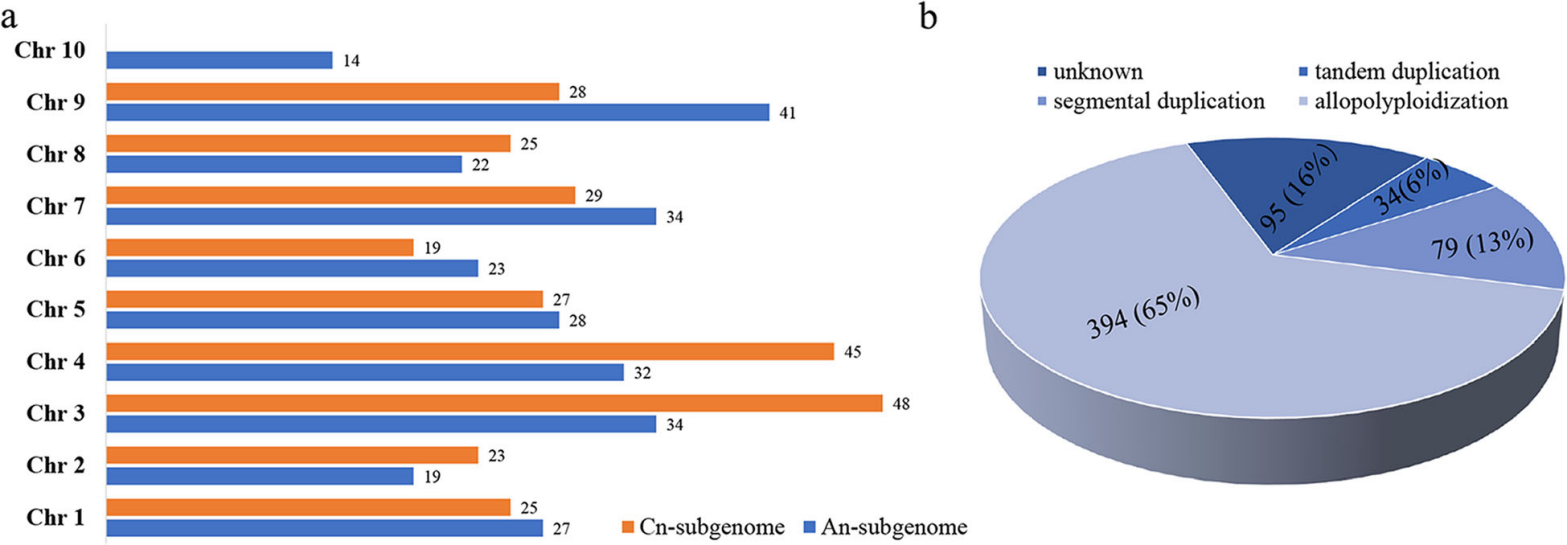
The distributions of candidate *BnabHLHs* on each chromosome and the expansion mechanism of *BnabHLHs*. **a** Distribution of *BnabHLHs* on the 19 *B. napus* chromosomes. The blue boxes indicate the chromosomes in the A_n_-subgenome, while the orange boxes indicate those in the C_n_-subgenome. The chromosome number is shown on the left and the number of *BnabHLHs* on a certain chromosome is listed on the right. **b** Percentage of *BnabHLHs* derives from the A_n_- or C_n_-subgenome

Chromosomal location analysis showed that there are 294 and 306 *BnabHLHs* in A_n_- and C_n_-subgenomes, respectively, indicating no biased tendency between these two subgenomes (Fig. 4a). The *BnabHLHs* are distributed on all the 19 *B. napus* chromosomes, but the genes on each chromosome are uneven within the two subgenomes. For example, in A_n_-subgenome, A10 has a minimum of 14 *BnabHLHs*, while A09 has a maximum of 41 genes. In C_n_-subgenome, C06 contains a minimum of 19 *BnabHLHs*, whereas C03 has as many as 48 genes. The average number of *BnabHLHs* in A_n_- and C_n_-subgenomes were 27.4 and 29.9, respectively.

Based on synteny analyses, 475 of the 602 *BnabHLHs* have syntenic relationships, 382 of which were inherited from *B. rapus* or *B. oleracea* genomes (Additional file 5: Table S5). In contrast, only 79 genes (7.8%) of 79 syntenic pairs underwent segmental duplication in the *B. napus* genome, and 34 genes (5.8%) from 28 syntenic pairs underwent tandem duplications (Fig. 4b). These results that the majority of *BnabHLHs* were obtained from allopolyploidy between *B. rapa* and *B. oleracea*. Accordingly, we found that the genome-wide duplicated genes make up the largest number of *BnabHLHs* in the majority of subfamilies. However, segmental duplicated genes make up the biggest number *BnabHLHs* in subfamilies S10 and S25 (nine genes per subfamily), and the tandem duplicated genes have the largest number in the S12 subfamily (11 genes). Furthermore, the *BnabHLHs* with intron pattern f expanded most in *B. napus* (31 duplicates), contributing to the largest proportion of *BnabHLHs* (Additional file 1: Table S1).

Taken together, the main expansion mechanism of *BnabHLHs* is whole-genome duplication (allopolyploidization), while segmental and tandem duplication events preferentially occurred in certain subfamilies with specific intron patterns.

### Expression profiles of *BnabHLHs* varied widely and were conserved within each subfamily

Gene expression patterns under different conditions can often give an indication of gene function. In order to explore the possible functions of the *BnabHLHs*, the temporal and spatial transcriptomes of the 602 *BnabHLHs* in 50 *B. napus* tissues of the root, leaf, flower, and seed at different developmental stages were characterized using RNA-seq (BioProject ID: PRJNA358784).

**Fig. 5:**
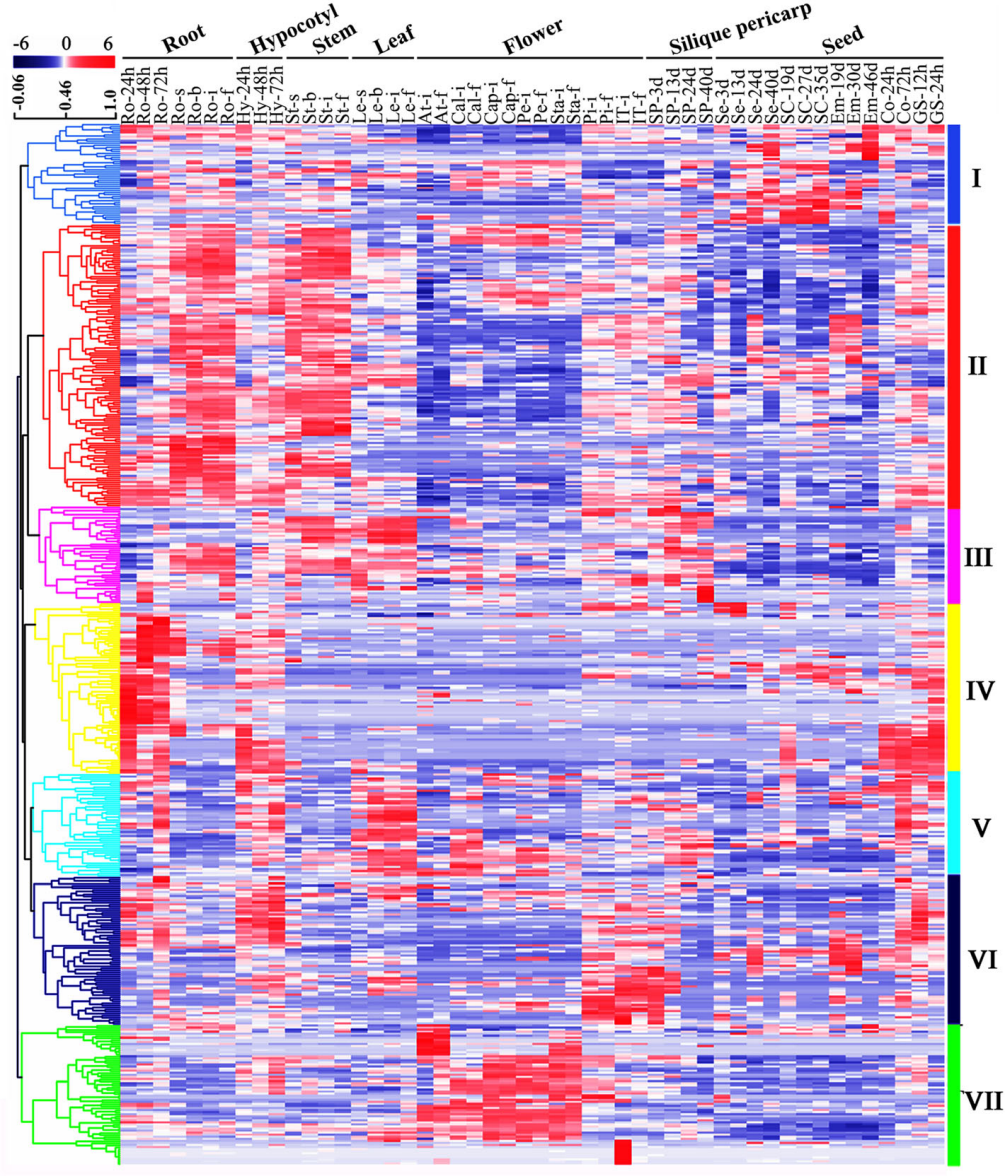
Expression profiles of candidate *BnabHLHs* in 50 *B. napus* tissues or organs across different developmental stages. Seven major blocks of different expression patterns are illustrated on the right. The tissues that were used for the expression analysis are indicated at the top of each column: GS, germinate seed; Hy, hypocotyl; Ao, anthocaulus; Ro, root; St, stem; Le, leaf; Cal, calyx; Cap, capillament; Pe, petal; Sta, stamen; Pi, pistil; IT, inflorescence tip; SP, silique; Se, seed; SC, seed coat; Em, embryo; Co, cotyledon. The ‘h’, ‘d’, ‘s’, ‘b’ ‘i’, ‘f’ indicate hour, day, seeding, budding, initial flowering, and full-bloom stages, respectively. The color bar represents log2 (FPKM ≥1)

A total of 47 *BnabHLHs* (7.79%) were excluded from our analysis with FPKM < 1, which may be pseudogenes or expressed only at specific developmental stages or under special conditions. The remaining genes (555 genes) have relatively high confidence expression levels (FPKM ≥1), the majority of which showed preferential expression in one or a few tissues/organs. Few genes were constitutively expressed in all tissues or organs tested, suggesting that this gene family tends to play regulatory roles at specific developmental stages or tissues. The wide expression profile of *BnabHLHs* suggests that they possess diverse roles in *B. napus*. The expression patterns of *BnabHLHs* were summarized into seven main blocks (I to VII) (Fig. 5). The *BnabHLHs* in blocks I to VII show obvious tissue-specific expression patterns: the genes in block I were highly expressed in seed coats; genes in block II were highly expressed in the root and stem tissues; genes in block III were mainly expressed in the vegetative organs e.g., root, stem, leaf and silique pericarp; the genes in block IV were primarily expressed in organs at the seedling stage, such as in the germinating seed (24 to 72 h), root, and hypocotyl; the genes in block V were mainly expressed in the pistil, silique pericarp, seed coat, and seed tissues; the genes in block VI had higher expression levels in the pistil and inflorescence tip tissues; whereas the genes in block VII were mainly expressed in flower tissues including capillament, petal, and stamen. Overall, there were 153, 136, 129, 137, 149 and 145 *BnabHLHs* having relatively high expression levels in the stem, hypocotyl leaf, silique pericarp, flower and seed respectively, while up to 301 genes were highly expressed in the roots, indicating that *BnabHLHs* may possess some previously unknown functions in the roots.

Typically, members of a given subfamily generally exhibit the same/similar expression profile. For example, members of the S2 subfamily were mainly expressed in the root and leaf at seedling, budding, and flowering stages (Additional file 6: Table S6). Moreover, the *bHLHs* in the same subfamily likely process the same or similar expression profiles across different species, and thus may share conserved functions during evolution. For example, *AtbHLH155/CPU* and *AtbHLH156/LHW* in the S23 subfamily play an essential role in establishing vascular cells and the size of vascular initial population in the root meristem [40]. The corresponding homologs in *B. napus* were also expressed in roots (Additional file 6: Table S6); their expression being relatively low in roots after germinating for 24 to 72 h but high in mature roots, which corresponds to their possible function in root vascular cells. Moreover, the expression profile of 28 pairs of tandem duplication genes, and 79 pairs of segmental duplication genes show similar expression patterns, indicating the functional redundancy of the duplicates (Additional files 5 and 6: Table S5 and S6).

Overall, the *BnabHLHs* were widely expressed but at different levels within *B. napus*. This was especially the case in the root, which provides an important clue as to the possible roles of this gene family. Moreover, *BnabHLHs* from a particular subfamily tend to possess conserved expression patterns and high structural similarity across different species, indicating a possible conservation of function during evolution.

### Many *BnabHLHs* were induced in hormone-treated roots

As discussed above, several *BnabHLHs* were highly expressed in the roots of *B. napus*, suggesting that they play a role(s) in some root-related biological process(es). To further explore their functional characteristics in roots, a comprehensive expression analysis of candidate *BnabHLHs* in roots treated with five hormones (IAA, auxin; GA_3_, gibberellin; 6-BA, cytokinin; ABA, abscisic acid and ACC, ethylene) was performed, based on previously acquired RNA-seq data (BioProject ID PRJNA608211).

Our results showed that many *BnabHLHs* (221, 36.7%) responded to more than one hormone treatment in roots, and most of these genes were clustered in ten subfamilies (S2, S16, S18, S19, S20, S21, S25, S27, S35, and S39) taking up over 50% of the genes in each subfamily (Additional file 7: Table S7). Meanwhile, the genes in 14 subfamilies (S1, S4, S5, S7, S9, S10, S12, S13, S14, S15, S24, S26, S34, and S38) were partly induced by the five hormone treatments. Interestingly, *BnabHLHs* in subfamilies S16, S18, and S21 were upregulated in roots under all five hormone treatments, while *BnabHLHs* in subfamilies S7, S23, and S34 were all downregulated. Notably, *BnabHLHs* in S12, which had low or no expression in roots (Fig. 5) were highly expressed after the five hormone treatments (Additional file 7: Table S7). Conversely, some genes that were normally highly expressed in roots were unresponsive to the hormone treatments. Indeed, *BnabHLHs* in S3, S11, S17, S22, S23, S28, S30, S31, S33, S36, and S37 had hardly any response to the hormone treatments in roots (Additional file 7: Table S7). Overall, most *BnabHLH* genes responded to hormone treatments in roots, indicating that they have important roles in hormone response in *B. napus* roots.

**Fig. 6:**
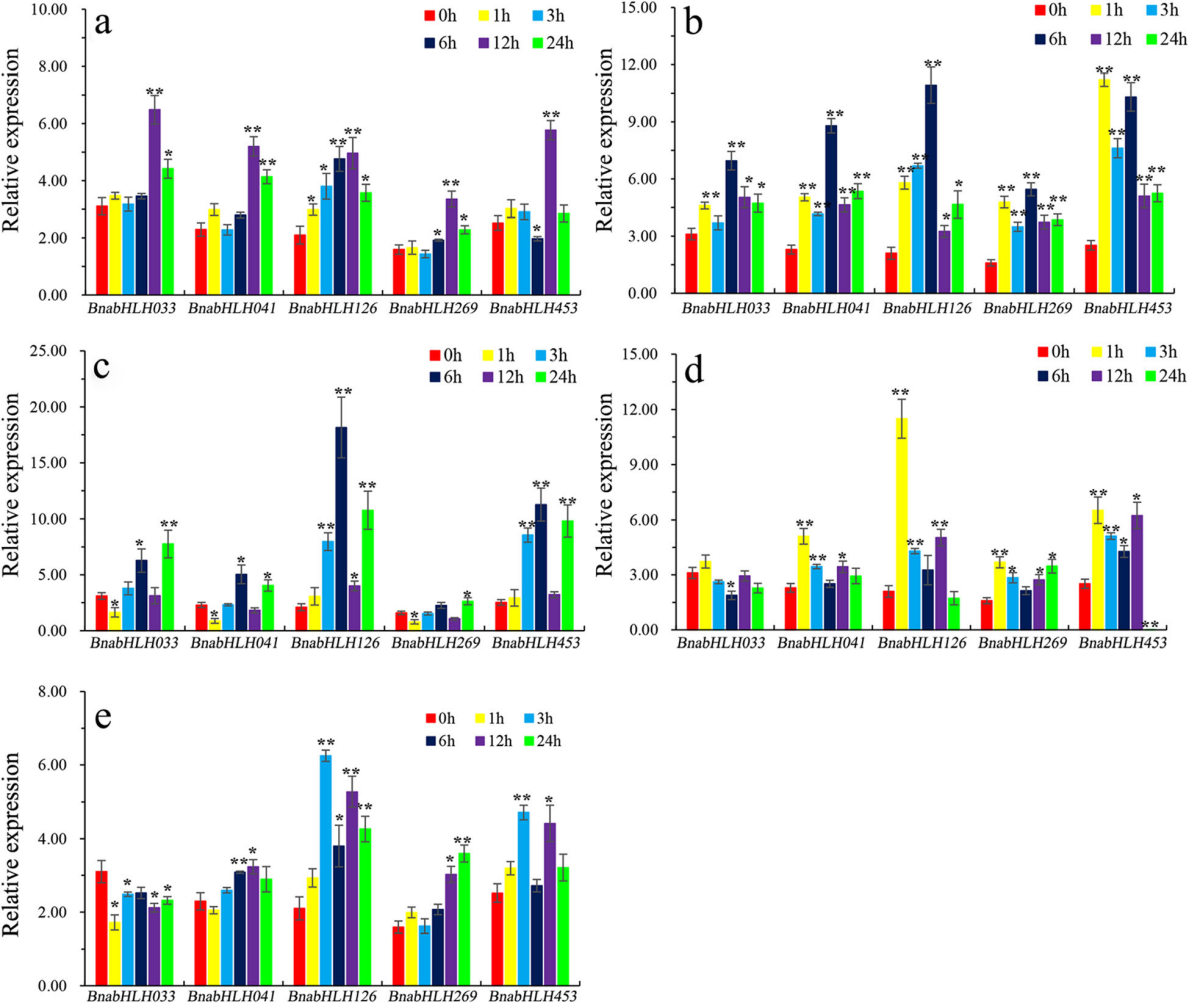
Expression of five *BnabHLHs* under hormone treatments. The transcript levels were determined in seedling roots by qRT-PCR under five different hormone treatments. **a** indole-3-acetic acid (IAA) treatment; **b** 1-aminocyclopropane-1-carboxylic acid (ACC) treatment; **c** abscisic acid (ABA) treatment; **d** gibberellic acid (GA_3_) treatment; **e** 6-benzyladenine (6-BA) treatment. Data are the mean ± standard deviation of three independent experiments. Error bars represent the standard errors from three independent replicates. Expression differences in *BnabHLHs* following hormone treatments was assessed by the Welch’s t-test (*P < 0.05; **P < 0.01). Comparison between treatments and control (CK, 0 h) according to Welch’s t-test (*P < 0.05; **P < 0.01)

To further verify the RNA-Seq results, five *BnabHLHs* that had high expression levels in roots (Additional file 6: Table S6) and obviously responded to hormone inductions (Additional file 7: Table S7) were selected to analyze their expression profiles under hormone inductions by qRT-PCR. Among them, three genes (*BnabHLH033*, *BnabHLH041,* and *BnabHLH269*) are orthologs of the *Arabidopsis ILR3* gene which was demonstrated to participate in auxin-conjugate metabolism [41], and two genes (*BnabHLH453* and *BnabHLH126*) are orthologs of the *Arabidopsis MYC2* gene which is involved in ABA, JA, and light signaling pathways. As shown in Fig. 6, our results by qRT-PCR were similar to that observed in the RNA-seq analyses. These five *BnabHLHs* were positively induced by all five hormone treatments, with *BnabHLH126* generally having a higher expression level than the others. Moreover, the genes from the same clade show similar expression patterns under certain hormone treatments. For example, *BnabHLH033*, *BnabHLH041,* and *BnabHLH269* from the *ILR3* clade in the S4 subfamily have similar expression patterns under IAA, ACC, and ABA treatments (Fig. 6a–c). However, their expression profiles are different under GA_3_ and 6-BA inductions, where *BnabHLH033* was significantly downregulated while *BnabHLH041* and *BnabHLH269* were significantly upregulated (Fig. 6d–e). Similarly, the expression of *BnabHLH453* and *BnabHLH126* from the *MYC2* clade in the S2 subfamily showed similar expression patterns under the five hormone treatments (Fig. 6). In addition, cis-acting elements analysis revealed that the promoter regions of these five *BnabHLHs* contain more than one cis-acting element that is related to the hormone response (Additional file 8: Table S8). This further supports our above results.

Together, our expression profile analyses revealed that a large proportion of *BnabHLHs* was induced by more than one hormone treatment in roots, indicating that this gene family may be important in root development in *B. napus*. Moreover, our qRT-PCR experiments confirmed the hormone-induced expression characteristics of *BnabHLH033*, *BnabHLH041*, *BnabHLH269*, *BnabHLH453,* and *BnabHLH126* which provided a valuable foundation for future functional research.

## Discussion

### Phylogenetic tree and subfamily division

As an important plant transcription factor super gene family, genome-wide analysis of *bHLHs* has already been conducted in a number of species [7–10]. However, we found that this gene family in plants still lacked a uniform subfamily classification. To date, there are two typical systematic subfamily divisions of this gene family in plants based on multiply species data, which were carried out by Pires and Dolan [6] and Carretero-Paulet et al. [7], respectively. Although both of these studies divided this gene family into 28 subfamilies, their results are in fact quite different, as only 19 subfamilies are the same between them while nine are different. The differences between these two divisions may result from the amount of *bHLHs* included in their phylogenetic analyses, as well as the criterion applied in the classification on the basis of the phylogenetic trees. In Pires and Dolan study [6], they adopted the *Arabidopsis* bHLHs proposed by Heim et al. [3] which included 118 *AtbHLHs*. However, there are a large number of bHLHs that were missed in this study due to the restriction of the *Arabidopsis* genome version used. By contrast, there was a total of 162 *AtbHLHs* in the study by Pires and Dolan [6] resulting in 12 newly identified orphan genes, such as *AtbHLH022/DYT1*, *AtbHLH159/P1r2,* and *AtbHLH102/BIM2* etc. Generally, the division of a gene family is based on the topology and bootstrap value of the phylogenetic tree [42, 43]. However, we found that subfamilies VII (a + b), IX, and IIIf in the results of Pires and Dolan [6] did not consist of a consensus node, but were across different branches/clades instead. By contrast, the situation was well defined in the results of Carretero-Pault et al. [7], indicating that these results may be more credible. Furthermore, most of the orphan genes (62 genes) identified by Pires and Dolan [6] were classified into different subfamilies by Carretero-Paulet et al. [7]. Consequently, an obvious decrease in the orphan gene proportions (total 15 genes, 2%) was observed, suggesting that their method offered a better solution for the classification of orphan genes. To date, this gene family has been characterized in various plants, such as *Z. mays* [8], tomato [9], and *B. rapa* [10], and the classification used in most of these studies referred to that of Pires and Dolan [6]. As a result, the subfamily classification in these studies was somewhat inadequate. For instance, in *Z. mays*, subfamilies VII, VIII, IX, XVI, and XVIII were not clustered in a consensus node [8]; the same situations were observed in *B. rapa* for subfamilies IIIf and Ib (2) [10]. Thus, we found that the criterion set forth by Carretero-Pault et al. [7] is more credible*.*

In this study, the 602 candidate *BnabHLHs* were classified into 35 subfamilies, using the method set forth by Carretero-Pault et al. [7] (Fig. 2). Among these subfamilies, one (S39) was newly identified (*AtbHLH151* homologs), and 11 were separated from five former subfamilies that had a relatively low bootstrap value in the study by Carretero-Pault et al. [7], including four subfamilies were separated into two new subfamilies (S5 and S33; S17 and S34; S21 and S35; S30 and S38) and one subfamily was divided into three new subfamilies (S24, S36, and S37) in our study. These differences may be attributed to the greater availability of sequences from *Brassicaceae* species being applied that have a close evolutionary relationship.

Several other genetic characteristics must be considered when performing subfamily classifications, including the presence of highly conserved intron patterns and motif distribution within each subfamily [42, 43]. In this study, the bHLH sequence characteristics within each subfamily were also highly conserved, which independently supports our phylogeny analysis and classification results. For example, subfamily S39 had a relatively high bootstrap value (Fig. 2a) and shared a conserved non-E-box and k intron pattern (Fig. 2b, c); subfamily S24, which was defined by Carretero-Pault et al. [7], was separated into subfamilies S24, S36, and S37 in this study (Fig. 2a), and these three new subfamilies contained a subfamily-specific DNA binding type, intron insertion patterns, and motifs (Fig. 2b, c); the former S17 subfamily was separated into S17 and S34 subfamilies in the present study (Fig. 2a), and the *BnabHLHs* in the new S17 subfamily had an E-box in the DNA binding domain while those *BnabHLHs* in S33 were all non-DNA binding types; similarly, the former S5 subfamily in the study by Carretero-Paulet et al. [7] was divided into two new subfamilies (S5 and S33) in our study as well.

Taken together, our study provides a more systematic classification of bHLH proteins in plants, which lays a good foundation for exploring the evolutionary characteristics of the bHLH gene family.

### The potential roles of *bHLHs* in *B. napus* roots

Given their crucial roles in diverse plant biological processes, research into the functional characteristics of plant bHLHs have garnered much attention in the past three decades. The majority of the known functions of *bHLHs* center on their roles in controlling the transcriptional networks of a number of biological processes, including metabolism and development. However, an increasing number of studies have demonstrated that they play important roles in plant root development as well. To date, the functions of *bHLHs* in plant roots mainly focused on their role in iron-uptake, salt and drought stress response, hormone response (e.g., responding to ABA, JA, BR, and IAA), and the regulation of the size of the vascular system in roots and meristems (Table 1).

**Table 1:** Functionally characterized plant bHLH proteins related to roots

Subfamily	Gene name	Species	Function
S1	*AtbHLH029/FIT*	*Arabidopsis*	Affects the plant’s iron uptake ability [44].
S2	*bHLH006/MYC2*	*Arabidopsis, Oryza sativa, Solanum lycopersicum, Marchantia polymorpha. Catharanthus roseus, Salvia miltiorrhiza, Triticum aestivum, Artemisia annua, Aquilaria sinensis*	Regulates the salt tolerance gene AtNHX6 and AtNHX6 in roots. Promotes ABA and JA responsiveness [45–49].
	*AtbHLH17/AtAIB*	*Arabidopsis*	Responds to NaCl, mannitol, and oxidative stress [50].
S3	*GmbHLHm1*	*Glycine max*	Linked to the activity of a unique class of ammonium channels and to signaling cascades influencing a nodule circadian clock [51].
S4	*AtbHLH11*	*Arabidopsis*	High expression in roots and acts as a negative regulator of iron homeostasis [52].
	*AtbHLH105/ILR3*	*Arabidopsis*	Regulates the network that controls wounding pathogen response in plant roots. Participates in auxin-conjugate metabolism [41, 53].
S5	*bHLH001/EGL3*	*Arabidopsis, Arabisalpina, Gossypium hirsutum, Petunia hybrida, Zea mays*	Enhancer of GL3.
	*bHLH002/GL3*		Partially redundantly regulates anthocyanin biosynthesis, trichome and root hair development [27, 28, 35, 54, 55].
S7	*AtbHLH92*	*Arabidopsis*	Responds to NaCl, dehydration, mannitol, and cold treatments, and regulates root elongation [56].
S12	*GmbHLH57 GmbHLH300 AtbHLH38 AtbHLH39 AtbHLH100 AtbHLH101 SlbHLH068*	*Arabidopsis, S. lycopersicum, O. sativa, Glycine max, Z. mays*	Induced by iron deficiency in both roots and shoots [44, 57–59].
S15	*AtbHLH112*	*Arabidopsis*	Regulates the salt stress response and root growth [60].
	*AtbHLH68*	*Arabidopsis*	Regulates ABA homeostasis and drought stress tolerance. Regulates lateral root development [61].
S16	*AtbHLH163/PRE6*	*Arabidopsis*	Negatively regulates auxin responses [62].
S19	*AtbHLH148/AIF2*	*Arabidopsis, O. sativa, S. lycopersicum*	Negatively regulate BR signaling pathways [63].
S23	*AtbHLH156/LHW*	*Arabidopsis*	Regulates the size of the vascular initial population in the root meristem [40].
S24	*AtbHLH024/SPT*	*Arabidopsis*	Regulates root growth by controlling the size of the root meristem [64].
S25	*AtbHLH74*	*Arabidopsis*	Regulates root growth in seedlings [65].
S26	*bHLH066/LRL1*	*Arabidopsis, Lotus japonicas, Physcomitrella patens, Z. mays, O. sativa, Hordeum vulgare.*	Act redundantly to positively regulate the development of root hairs [66–68].
	*bHLH069/LRL2*		
	*bHLH082/LRL3*		
	*bHLH007/LRL4*		Negatively controls root hair growth [67].
	*bHLH059/LRL5*		
	*ZmPTF1*	*Z. mays*	Regulates drought tolerance in maize by promoting root development and ABA synthesis [69].
S27	*AtbHLH129*	*Arabidopsis*	Regulates root elongation and ABA response [50].
	*bHLH122*	*Arabidopsis, O. sativa, S. lycopersicum*	Regulates the salt tolerance gene AtNHX6 in roots. Promotes ABA and JA responsiveness [45].
S28	*bHLH086/RSL1*	*Arabidopsis, M. polymorpha, Brachypodium, P. patens*	Partially redundant and involved in root hair development [70–74].
	*bHLH085/RSL2*		
	*bHLH084/RSL3*		
	*bHLH054/RSL4*		
	*bHLH139/RSL5*		

As mentioned above, the bHLHs are widely expressed in root tissues in *B. napus*. Accordingly, many members in nine subfamilies of this gene family (S5, S7, S15, S23–S28) have been identified to regulate many root processes (Table 1). For example, *AtbHLH156/LHW* from subfamily S23 plays an essential role in establishing vascular cells and the size of the vascular initial population in the root meristem [40]. Additionally, *AtbHLH024/SPT* in S24 regulates root growth by controlling the size of the root meristem [64]. Homologs of *AtbHLH002/GL3* (S5), *AtLRLs* (S26), and *AtRSLs* (S28) are also involved in root hair development [35, 54, 66, 67, 69–75]. Moreover, *AtbHLH92* in S7 and *AtbHLH129* in S27 regulate root elongation [50, 56], while *AtbHLH74* of S25 regulates seedling root growth [65]. In this study, we found that the *BnabHLH* orthologs of those functionally characterized *bHLHs* were highly expressed in *B. napus* roots (Fig. 5), indicating their potential importance in root development.

In this study*, bHLHs* were also shown to be involved in hormone signaling pathways and environmental stress in plant roots. To date, many members in 11 subfamilies (S1-S4, S7, S12, S15, S16, S19, S26, and S27) are involved in plant responses to iron, salt, and drought stresses, and are involved in ABA and JA pathways (Table 1). For example, members in subfamilies S1, S4, and S12 affect iron uptake [44, 52, 57]. Studies have also found that *AtbHLH17/AIB* (S2), *AtbHLH92* (S7), *AtbHLH112* (S15), and *bHLH122* (S27) respond to salt stress [45, 56, 60, 76], while *AtbHLH68* (S15) and *ZmPTF1* (S26) respond to drought stress [61, 69]. Meanwhile, many *MYC2* homologous genes in the S2 subfamily (e.g., *Catharanthus roseus CrMYC2* [46] and *Salvia miltiorrhiza SmMYC2* [47]) are known to be involved in cellular responses to ABA and JA [20–22]. Similarly, members in subfamilies S15 and S26 have been demonstrated to respond to ABA [61, 69], while members of the S19 subfamily negatively regulate the BR signaling pathway [63]. Additionally, genes in the S4 subfamily participate in auxin-conjugate metabolism [41, 53] (Table 1). Accordingly, our RNA-seq data shows that many *BnabHLHs* (221, 36.7%) respond to more than one hormone treatment in *B. napus* roots (Additional file 7: Table S7). Our qRT-PCR analysis further confirmed that two *BnabHLHs* (*BnabHLH126* and *BnabHLH453*) in S2 and three *BnabHLHs* (*BnabHLH033*, *BnabHLH041,* and *BnabHLH269*) in S4 were induced by hormone treatments in *B. napus* roots (Fig. 6). Together, these results suggest that bHLHs have an important role in hormone signaling in plant roots.

Taken together, expression profile analyses along with previous gene function research indicates that *BnabHLHs* may be integrally involved in *B. napus* root biological processes including root growth and hormone signaling. Our study provided a valuable foundation for further *bHLH* gene function research.

## Conclusion

In this study, 602 *BnabHLHs* were identified from *B. napus* genome and were classified into 35 subfamilies. Eight conserved intron insertion patterns were observed in the bHLH domains of BnabHLHs. Members of the same subfamily generally have conserved gene structure and protein motif composition. Allopolyploidization between *B. rapa* and *B. oleracea* is the major driving force for large gene expansion of bHLH genes in *B. napus* genome. The *BnabHLHs* have diverse expression profiles in 50 *B. napus* tissues at different developmental stages, but members in the same subfamily generally have a similar expression pattern. Moreover, many *BnabHLHs* have relative high expression levels in *B. napus* roots. Many *BnabHLHs* (about 37%) were hormone-inducible in *B. napus* roots by RNA-Seq analyses. Among them, the hormone-induced expression characteristics of five genes, *BnabHLH033*, *BnabHLH041*, *BnabHLH269*, *BnabHLH453,* and *BnabHLH126* were confirmed by qRT-PCR assay. Overall, this study provides important clues about the potential functions of the *BnabHLHs* that will be useful for gene function research in the future.

## Methods

### Sequence retrieval

The sequences of 167 *Arabidopsis* bHLHs proteins (AtbHLHs) were retrieved from the TAIR (http://www.arabidopsis.org/). To identify bHLH genes in the *B. napus* genome, we referred to the previously described method of Guo et al. [29] with minor modifications. In brief, we performed a repeated BLASTP search (e values of < 1.0) against the proteome of *B. napus* (Darmor–*bzh* ecotype) in GENOSCOPE (http://www.genoscope.cns.fr/brassicanapus/) [30], using at least one representative protein sequence of the bHLH domain for each bHLH subfamily [7] as queries. Each matching sequence was then used to search the *B. napus* genome database, until no new sequences were found. The redundant sequences were discarded according to the loci in the genome. To ensure the integrity of the bHLH gene data in *B. napus*, we also searched another sequenced *B. napus* cultivar genome (ZS11 ecotype) in the NCBI database (http://www. ncbi.nlm.nih.gov/genome/annotationeuk/Brassica_napus/101/) [31]. We then confirmed the putative non-redundant sequences to ensure that the candidates contained the bHLH domain using ExPASy (http://expasy.org/prosite/) [77] and MEGA 5.0 [78] software. The sequence information of the candidate genes in these two ecotypes was manually compared and corrected. Finally, all candidates were named according to their chromosome locus. Similarly, the candidate bHLH genes in the *B. oleracea* genome (v1.0) were identified by the same method in the BRAD database (http://brassicadb.org/brad/).

### Phylogenetic tree construction

Multiple sequence alignment of the bHLH domains of 167 *Arabidopsis* (AtbHLHs) and 602 *B. napus* (BnabHLHs) bHLH proteins was carried out by MAFFT online software under default parameters (http://mafft.cbrc.jp/alignment/server/) [79]. Based on the result of multiple sequence alignment, a NJ tree was constructed using MEGA 5.0 [78] with a bootstrap test (1000 replicates) based on p-distance model and pairwise deletion for gap treatment. Tree file was visualized using FigTree v1.3.1 (http://tree.bio.ed.ac.uk/software/figtree/).

### Chromosome localization and gene synteny analysis

The information of chromosome length and bHLH genes locations were acquired from the BRAD (http://brassicadb.org/brad/index.php) and Genoscope databases (http://jacob.cea.fr/drf/ifranc oisjacob/Pages/Departements/Genoscope.aspx) [30, 80], respectively. The chromosome map of candidate *BnabHLHs* was drawn by Mapchart software. Similarly, the chromosome locations of *bHLHs* in *Arabidopsis*, *B. rapa*, and *Brassica oleracea* genomes were analyzed by the same method. Gene synteny analysis of *bHLHs* in *Arabidopsis*, *B. napus*, *B. rapa*, and *B. oleracea* genomes was performed by CoGe online software (https://genomevolution.org/coge/) [39]. Accordingly, gene replication events for all candidate *bHLH* genes were analyzed based on the syntenic blocks for intra- and inter-genomic comparisons. Tandem duplication genes were identified according to their physical locations within individual chromosomes and < 1 intervening gene.

### Intron/exon structure analysis

The corresponding CDS and DNA sequences of candidate bHLH genes in *B. napus* were obtained from Genoscope database as well. The intron distribution, position and phase of candidate *B. napus* bHLH genes, including exon and intron numbers were analyzed by the Gene Structure Display Server (GSDS) online tool with default parameters (http://gsds.cbi.pku.edu.cn/) [38]. We manually located the intron insertion sites in the corresponding protein sequences as well.

### Identification of conserved motifs

To identify the conserved protein motifs outside the bHLH domain, the protein sequences of candidate *BnabHLHs* were analyzed using MEME Version 5.1.1 software (http://meme-suite.org/tools/meme) [81]. The parameters settings are: number of motifs to find, 30; minimum width of motifs, 6; and maximum width of motifs, 250.

### Expression analysis of *BnabHLHs*

The *B. napus* expression datasets were downloaded from the BioProject (NCBI database: PRJNA358784). The data were obtained from various tissues at different *B. napus* developmental stages and under the induction of five different hormones (IAA, ABA, 6-BA, ACC, and GA_3_). The expression profiles of *BnabHLHs* were analyzed using the MeV v4.9 software (https://sourceforge.net/projects/mev-tm4/files/) [82] with the HCL method, and the heatmaps were drawn using the R package; The tissue high expression genes identified by z-score above3 in at least one of the organization samples [82]. All genes with FPKM < 1 were excluded from the heatmap, as they may be pseudogenes or may be expressed only under specific stresses or treatments.

For qRT-PCR analysis, seeds of *B. napus* variety ZS11 were obtained from the College of Agriculture and Biotechnology, Southwest University, and germinated on petri dishes. At the five-leaf stage, seedlings were treated in Hoagland liquid medium containing one of five phytohormones (50 μM ABA, 120 μM GA, 75 μM 6-BA, 60 μM ACC, and 10 μM IAA). The seedlings were then grown in an artificial climate chamber at 25 °C with a 16/8 h photoperiod (day/night). The root tissues were then harvested at 0, 1, 3, 6, 12, and 24 h after the treatments and immediately frozen in liquid nitrogen and stored at − 80 °C for RNA isolation. Extractions of total RNA and subsequent cDNA synthesis were performed as described by Guo et al. [29]. *B. napus Actin7* (*BnActin7*) (GenBank accession no. AF024716) was used as the internal control. The SYBR-Green PrimeScript RT-PCR Kit (Takara, Dalian, China) was used for qRT-PCR amplification in a CFX Connect™real-time PCR system (Bio-Rad, Chongqing, China). The reaction conditions for real-time PCR were as follows: initial denaturation at 95 °C for 3 min, followed by 40 cycles of denaturation at 95 °C for 10 s and annealing at 58 °C for 20 s. The relative expression levels of candidate genes were determined using the 2^−ΔΔCt^ method [83]. Each treatment consists of three technical replicates. Expression levels were calculated as the mean signal intensity across the three replicates. The primers used in this analysis are listed in Additional file 9: Table S9.

## Supplementary information

**Additional file 1: Table S1.** Features of the 602 *bHLH* genes from *Brassica napus* identified in this study.

**Additional file 2: Table S2.** Identification of the 245 *bHLH* genes from *Brassica oleracea* in this study.

**Additional file 3: Table S3.** Amino acid composition of the bHLH domain across different species.

**Additional file 4: Table S4.** Conserved motifs identified in different bHLH subfamilies in this study.

**Additional file 5: Table S5.** Syntenic relationships of *bHLH* genes in *Brassica napus*, *Brassica rape,* and *Brassica oleracea*.

**Additional file 6: Table S6.** The expression values of *BnabHLHs* in 50 *Brassica napus* tissues across different developmental stages.

**Additional file 7: Table S7.** The expression values of *BnabHLHs* under different hormone treatments.

**Additional file 8: Table S8.** Cis-acting element analysis of *BnabHLH* promoter sequences

**Additional file 9: Table S9.** List of primers used for the real-time PCR analysis.

## Data Availability

The RNA-Seq datasets used in the current study are available in the Sequence Read Archive (SRA) at NCBI (SRA accession: PRJNA574049 and PRJNA608211) repository.

## Abbreviations

6-BA: Cytokinin
ABA: Abscisic acid
ACC: Ethylene
bHLH: Basic helix-loop-helix
FPKM: Fragments per kilobase of transcript per million fragments mapped
GA_3_: Gibberellin
GSDS: Gene Structure Display Server
IAA: Auxin
NJ: Neighbour-Joining
qRT-PCR: Quantitative real time polymerase chain reaction
